# Clinical Outcomes and Biomechanical Evaluation of the Cement-Catching Screw Technique for Osteoporotic Vertebral Fractures

**DOI:** 10.7759/cureus.85955

**Published:** 2025-06-13

**Authors:** Hisakazu Shitozawa, Haruo Misawa, Yoshiaki Oda, Ryoji Joko, Masaya Takahashi, Koji Uotani, Yasuyuki Shiozaki, Tomoko Tetsunaga, Kensuke Shinohara, Ryo Nakamichi, Masataka Ueda, Ryo Takatori, Kazutaka Yamashita, Toshifumi Ozaki

**Affiliations:** 1 Department of Orthopaedic Surgery, Science of Functional Recovery and Reconstruction, Okayama University Graduate School of Medicine, Dentistry and Pharmaceutical Sciences, Okayama, JPN; 2 Department of Orthopaedic Surgery, Ryusou Orthopaedic Hospital, Okayama, JPN; 3 Department of Orthopaedic Surgery, Faculty of Medicine, Dentistry and Pharmaceutical Sciences, Okayama University, Okayama, JPN; 4 Department of Orthopaedic Surgery, Japanese Red Cross Okayama Hospital, Okayama, JPN; 5 Department of Orthopaedic Surgery, Okayama University Hospital, Okayama, JPN; 6 Department of Orthopaedic surgery, Mitoyo General Hospital, Okayama, JPN; 7 Department of Sports Medicine, Faculty of Medicine, Dentistry and Pharmaceutical Sciences, Okayama University, Okayama, JPN

**Keywords:** balloon kyphoplasty, cement-catching screw, intermediate screws, osteoporotic vertebral fractures, pullout strength

## Abstract

Objectives: We developed a cement-catching screw (CCS) technique for pedicle screw insertion into hardened cement, connecting anterior and posterior vertebral elements during balloon kyphoplasty (BKP) for osteoporotic vertebral fractures (OVFs). This study reports the CCS technique, clinical outcomes, and biomechanical properties.

Methods: This retrospective study included 59 patients (20 men, 39 women; mean age, 77.4 ± 8.7 years) who underwent BKP with one-above-one-below posterior fixation for OVFs between 2020 and 2023. Patients were divided into CCS (−) (without intermediate screws, n = 28) and CCS (+) (with intermediate CCSs, n = 31) groups. Clinical and radiographic outcomes, including activities of daily living, vertebral wedge angle (VWA), surgical level Cobb angle (CA), anterior vertebral body height (AVBH), screw loosening, pullout, and adjacent vertebral fractures, were evaluated preoperatively, postoperatively, and at the final follow-up (≥6 months). Biomechanical pullout strength was assessed at different insertion depths (5, 10, and 15 mm) using polymethylmethacrylate cement.

Results: No significant differences were observed between groups in age, sex, follow-up duration, or blood loss; however, the operation time was significantly longer in the CCS (+) group than in the CCS (−) group (P < 0.0001). Radiographic outcomes showed no significant differences in the VWA, CA, AVBH, adjacent vertebral fracture rates, and reoperation rates. However, the incidence of adjacent pedicle screws loosening and pullout was significantly higher in the CCS (−) group than in the CCS (+) group (P = 0.046 and 0.0084, respectively). The correction loss of the CA was significantly lower in the CCS (+) group (CCS (−), 5.6° ± 4.8°; CCS (+), 3.5° ± 4.8°, P = 0.023). The biomechanical test revealed pullout strengths of 683 ± 164, 2231 ± 208, and 3477 ± 393 N for insertion depths of 5, 10, and 15 mm, respectively, with significant increases by depth (P = 0.003 and 0.009).

Conclusions: The CCS technique improves anterior-posterior vertebral body stability, enhances fixation strength, and contributes to better surgical outcomes in OVFs treatment.

## Introduction

Surgical treatment for osteoporotic vertebral fractures (OVFs) aims to relieve pain, provide spinal support, correct kyphotic deformity, and prevent further spinal deformity [1]. Many patients with OVFs are elderly with multiple comorbidities; therefore, minimally invasive treatment is desirable [2]. Balloon kyphoplasty (BKP) is widely used, showing favorable outcomes by minimally invasively correcting vertebral wedge deformities, reconstructing the anterior column, and improving vertebral body instability [3-6]. However, in split-type fractures where the vertebral body is divided anteroposteriorly or in the presence of pedicle fractures, anterior cement migration becomes a significant problem, leading to unsatisfactory outcomes with BKP alone [7]. Furthermore, in cases of intervertebral instability or failure of the posterior ligamentous complex, BKP alone is challenging, often resulting in loss of correction and progression of kyphotic deformity. These cases may require posterior fixation using pedicle screws (PS) inserted into the adjacent vertebrae, one level above and below the fractured vertebra [8]. In particular, when the anterior and posterior vertebral elements are separated, vertebra-pediculoplasty can be performed, inserting cannulated screws during BKP to connect the separated anterior and posterior columns, thereby preventing anterior cement migration [9]. In addition, for osteolytic metastatic tumors or severe OVFs, stent screw-associated internal fixation (SAIF) has shown favorable outcomes. This technique combines vertebral body stenting with cannulated screws to connect the anterior and posterior elements [10]. Furthermore, a finite-element model (FEM) analysis of SAIF demonstrated that it reduces the stresses in the anterior and middle columns, which may help prevent refracture and progression of vertebral collapse [11]. However, both techniques involve inserting screws before the cement hardens, which may result in the screws becoming firmly bonded to the cement, making their removal challenging.

To address this issue and prevent screw-cement adhesion, a cement-catching screw (CCS) technique was developed. In this method, a pilot hole is created during BKP to harden the cement. Once the cement has fully hardened, the hole is tapped and the screw is subsequently inserted. This technique prevents screw adhesion to the cement, making its removal and reinsertion easier. The CCS technique is mainly characterized by its ability to enhance the anteroposterior stability by gripping the cement within the anterior column. Additionally, anchoring into the cement, which is stronger than the bone, increases the screw fixation strength. In posterior fixation for fractured vertebrae, screws inserted into the fractured vertebra (intermediate screws, IMS) can enhance fixation. The CCS technique uses conventional spinal implant PS; thus, they can be integrated into posterior fixation constructs and used as IMS, potentially strengthening short-segment constructs.

This study describes the newly developed CCS technique and evaluates its clinical outcomes, highlighting its potential to enhance short-segment construct stability. Furthermore, this study investigated the biomechanical characteristics of CCS, demonstrating its ability to enhance anteroposterior stability. Compared with conventional techniques, CCS optimizes short-segment fixation strategies, reducing implant failure and the risk of postoperative collection loss. Through clinical and biomechanical analyses, this research aims to provide valuable insights into the advancement of minimally invasive spinal fixation techniques, ultimately improving treatment strategies for patients with OVFs.

## Materials and methods

Surgical technique

**Figure 1:**
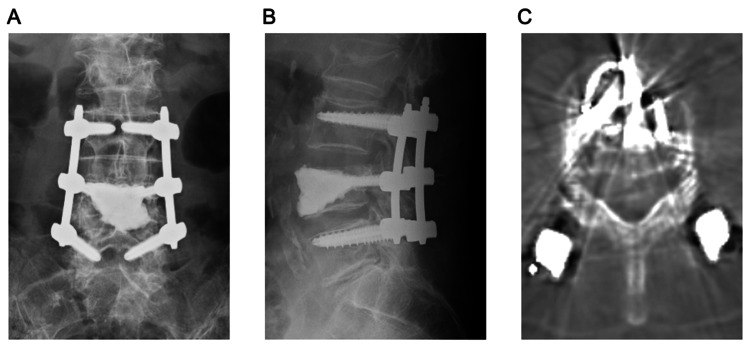
CCS placement CCS were inserted into the L4 vertebra and connected with pedicle screws positioned one level above and one level below. (A) Anteroposterior radiograph, (B) lateral radiograph, and (C) computed tomography axial view of the CCS. CCS, cement-catching screws.

**Figure 2:**
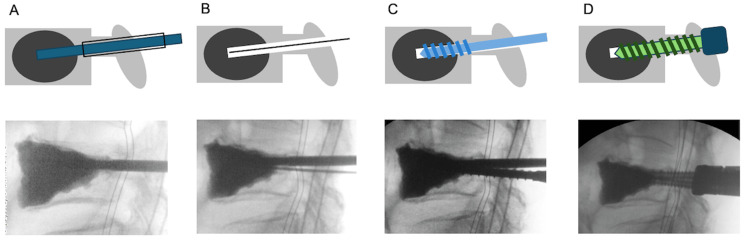
Surgical technique (A) Creating a pilot hole: After completing the standard balloon kyphoplasty procedure, the bone filler device (diameter, φ3.2 mm) is inserted into the cement to maintain a pre-hole for screw insertion until the cement hardens. (B) Guidewire technique: Once the cement has hardened, the filler is replaced with a guidewire. (C) Tapping: Tapping with instruments of increasing diameters (φ4.5 and φ5.5 mm) to prepare the path for screw insertion. (D) Screw insertion: A screw is inserted so that its tip reaches the hardened cement.

Under general anesthesia, patients were placed prone on a radiolucent bed. Ballooning and cement injection were performed bilaterally under fluoroscopic guidance, following the standard BKP procedure. After cement injection, the bone filler device (φ3.2 mm) was deeply inserted into the cement and rotated to prevent adhesion until the cement hardened, thereby creating a pilot hole. Once the cement had fully hardened, the device was replaced with a guidewire, and tapping was sequentially performed from φ4.5 mm to φ5.5 mm. Finally, the φ5.5 mm screw was inserted to anchor into the cement (Figures 1, 2). Early mobilization and rehabilitation were initiated the next day based on the patient’s pain level. No specific restrictions were imposed on daily activities.

Clinical study

This retrospective study included 59 patients (20 men and 39 women; mean age, 77.4 ± 8.7 years) who underwent BKP for OVFs (Th11-L4) combined with posterior fixation at one level above and below the fractured vertebra between 2020 and 2023. The inclusion criteria were as follows: (i) diagnosed with OVFs requiring surgery, (ii) underwent BKP combined with posterior fixation, (iii) age ≥60 years, and (iv) followed up for at least six months after surgery. Patients were excluded if they met any of the following criteria: (i) presence of pathological fractures caused by malignancy or infection, (ii) presence of neurological deficits requiring decompression surgery, (iii) history of prior surgery at the affected level, and (iv) incomplete medical records or insufficient data for analysis.

Patients were classified into two groups: CCS nonuse group (CCS (−), BKP and fixation without IMS) and CCS use group (CCS (+), BKP with fixation using intermediate CCS). Lateral spine radiographs taken preoperatively, postoperatively, and at the final follow-up (≥6 months) were analyzed for vertebral wedge angle (VWA, angle between the upper and lower endplates of the fractured vertebra), surgical-level Cobb angle (CA, the angle formed between the upper endplate of the cephalad adjacent vertebra and the lower endplate of the caudal adjacent vertebra), and anterior vertebral body height (AVBH, the ratio of the anterior height of the fractured vertebra to the average anterior height of the adjacent vertebrae), based on the measurement method described by Wang et al. [12]. In addition, screw loosening, pullout, and adjacent vertebral fractures were evaluated using radiographs and computed tomography scans at the final follow-up. Loosening was defined as ≥1 mm radiolucency around screws, whereas pullout was identified as a halo sign and axial displacement of the screw compared with immediate postoperative images [13]. Patients’ activities of daily living (ADLs) and performance status were assessed pre- and postoperatively at six months. Patient demographic information was retrospectively collected from medical records.

This study was approved by the Ethics Committee of Okayama University (Permission No. 2111-043), and informed consent was obtained from all patients prior to their participation.

Biomechanical study: Axial pullout test

**Figure 3:**
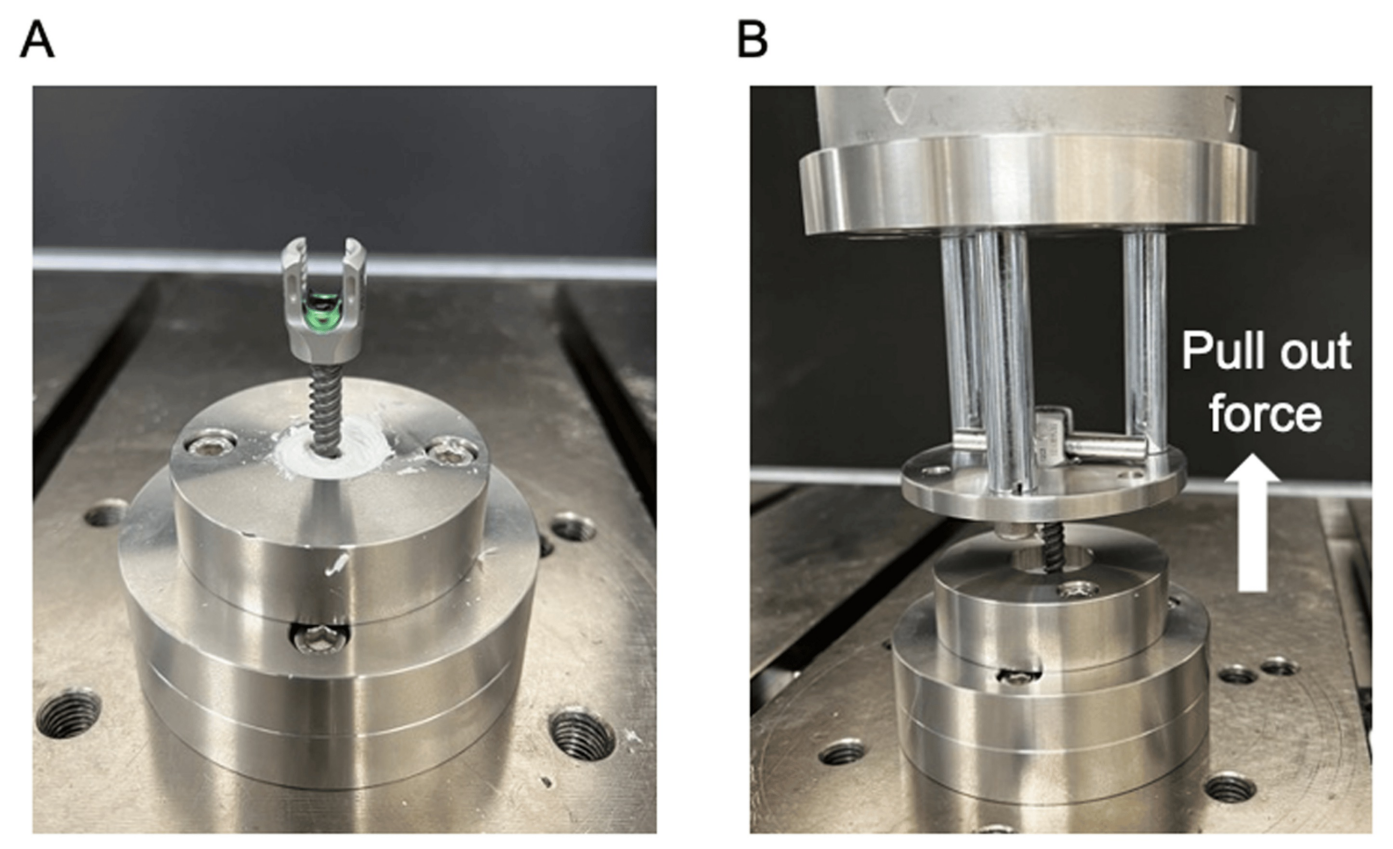
Procedure for the axial pullout test (A) Screw insertion: A pilot hole is created following the same technique used in clinical practice. After the cement has completely hardened, screws of varying lengths (5, 10, and 15 mm) are inserted into the pilot hole. (B) Testing machine connection: The inserted screw is connected to the testing machine (ElectroPuls E10000; Instron, Canton, MA) via a rod and a set screw, enabling the setup for the axial pullout test.

Polymethylmethacrylate (PMMA) cement (Kyphon HV-R Bone Cement, Medtronic, Dublin, Ireland, Cat. #C01A) was prepared at room temperature and poured into a specially designed jig. Before the cement had fully hardened, a bone filler device was inserted perpendicularly to create a pilot hole. The inserted bone filler device was rotated carefully for over 20 min to prevent adhesion. It was then replaced with a guidewire and tapped sequentially from φ4.5 to φ5.5 mm. Moreover, φ5.5 × 35 mm multiaxial PS (CD Horizon Solera, Medtronic, Cat. #55840005535) were marked at 5, 10, and 15 mm from the tip. The screws were inserted to engage the planned depths, and axial pullout tests were performed using an ElectroPuls E10000 (Instron, Canton, MA) testing machine in accordance with the American Society for Testing and Materials F543-17 at a displacement rate of 1 mm/min (Figure 3). Pullout force and displacement data were recorded at 100 Hz using Wave Matrix2 (Instron). Each depth was tested three times, and the maximum pullout strength was obtained by defining failure as the point at which the pullout force on the force-displacement curve began to decrease.

Statistical analysis

Data were analyzed using GraphPad Prism version 10.2.0 (GraphPad Software, San Diego, CA). Distributions of variables are presented as mean and standard deviation. The Mann-Whitney test was performed to compare continuous variables between two groups, whereas one-way analysis of variance was employed for comparisons among three groups. Fisher’s exact test and chi-square test were applied for categorical data analysis. Significance was defined as P <0.05.

## Results

Clinical evaluation

The distribution of fracture levels was as follows: Th11 in 12% (n = 7), Th12 in 51% (n = 30), L1 in 25% (n = 15), L2 in 3% (n = 2), L3 in 2% (n = 1), and L4 in 7% (n = 4). The timing of surgery was categorized into acute fractures (within three months after injury) in 53% (n = 31) and chronic fractures (beyond three months after injury, including pseudarthrosis) in 48% (n = 28). The fracture types included intervertebral instability caused by endplate fractures in 46% (n = 27), pedicle fractures in 40% (n = 24), split-type fractures in 7% (n = 4), and vertebra plana in 7% (n = 4). Among these, 7% (n = 4) had neurological symptoms such as lower limb pain or incomplete paralysis.

In this study, 28 and 31 cases were classified into the CCS (−) and CCS (+) groups, respectively. No significant differences were observed in terms of age, sex, follow-up duration, or intraoperative blood loss. However, the CCS (+) group had a significantly higher proportion of chronic fractures (P < 0.03) and longer operative times (P < 0.0001). Preoperatively, approximately half of the patients in both groups were bedridden; however, by the final follow-up, all patients were able to mobilize. Patients were divided into two groups based on the performance status (<2 and ≥3), with no significant differences observed, regardless of CCS use (Table 1).

**Table 1:** Clinical features of patients ADL, activities of daily living; PS, performance status. *Statistical significance is defined as P <0.05. ^#^Indicates chi-square test. ^##^Indicates Fisher’s exact test. All other comparisons used the Mann-Whitney test. Values are presented as mean ± standard deviation or counts with percentages.

	CCS (-), n = 28	CCS (+), n = 31	P-value (test statistics)
Age (years)	78.7 ± 6.8	76.3 ± 10.0	0.45 (U = 384)
Sex	Male: 9, female: 19	Male: 11, female: 20	0.79^#^ (chi-square = 0.073, df = 1)
Follow-up duration (months)	16.9 ± 8.9	15.5 ± 6.9	0.80 (U = 417)
Fracture type	Acute: 19, chronic: 9	Acute: 12, chronic: 19	<0.03*^,#^ (chi-square = 5.0, df = 1)
Operation time (min)	49.2 ± 10.3	83.2 ± 18.6	<0.0001* (U = 40)
Blood loss (mL)	40.0 ± 20.9	54.8 ± 38.4	0.13 (U = 341)
Pre-operation ADL, n (%)			0.03*^,^^##^
Bed	13 (46%)	14 (45%)	
Wheel chair	1 (4%)	3 (10%)	
Walk with a walker	8 (28%)	1 (3%)	
Walk with a cane	3 (11%)	0 (0%)	
Post-operation ADL, n (%)			>0.99^##^
Bed	0 (0%)	0 (0%)	
Wheel chair	1 (4%)	1 (3%)	
Walk with a walker	5 (18%)	6 (19%)	
Walk with a cane	5 (18%)	5 (16%)	
Pre-operation PS, n (%)			0.71^#^ (chi-square = 0.14, df = 1)
PS <2	14 (45%)	14 (50%)	
PS ≥3	17 (55%)	14 (50%)	
Post-operation PS, n (%)			0.94^##^
PS <2	30 (97%)	27 (96%)	
PS ≥3	1 (3%)	1 (4%)	

Radiographically, no significant intergroup differences were observed in terms of VWA, CA, AVBH, adjacent vertebral fracture, or reoperation rates across timepoints. However, loosening of the PS inserted into the adjacent vertebrae occurred significantly more frequently in the CCS (−) group (CCS (−), 39% (n = 11); CCS (+), 16% (n = 5), P = 0.046). In addition, vertebral fractures at the PS insertion sites were observed in two cases in the CCS (−) group and five in the CCS (+) group, which resulted in screw loosening. Screw pullout also occurred significantly more frequently in the CCS (−) group (CCS (−), 21% (n = 6), CCS (+), 0% (n = 0), P = 0.0084), with three of the six cases requiring the extension of the posterior fixation. In contrast, CCS loosening was not observed in the CCS (+) group, and even when loosening occurred in the adjacent PS, pullout did not occur. Posterior fixation extension was performed in one case because of an adjacent vertebral fracture outside the fixation range. Although no significant difference was found in the loss of correction for the VWA and AVBH at the final follow-up, the loss of correction in the CA was significantly lower in the CCS (+) group than in the CCS (−) group (CCS (−), 5.6° ± 4.8°; CCS (+), 3.5° ± 4.8°, P = 0.023). When excluding cases with fractures at the superior and inferior vertebrae of the fixation range, the reduced loss of correction in the CCS (+) group became even more pronounced (CCS (−), 5.0° ± 4.6°; CCS (+), 1.9° ± 3.1°, P = 0.0012) (Table 2).

**Table 2:** Radiographic outcomes VWA, vertebral wedge angle; CA, surgical-level Cobb angle; AVBH, anterior vertebral body height; PS, pedicle screw. *Statistical significance is defined as P <0.05. ^#^Indicates chi-square test. ^##^Indicates Fisher’s exact test. All other comparisons used the Mann-Whitney test. Values are presented as mean ± standard deviation or counts with percentages.

	CCS (-)	CCS (+)	P-value (test statistics)
VWA (°)			
Pre-operation	17.3 ± 6.9	21.0 ± 7.4	0.064 (U = 299)
Post-operation	10.3 ± 5.4	11.5 ± 5.6	0.54 (U = 394)
At final follow-up	11.0 ± 5.5	12.2 ± 5.7	0.51 (U = 391)
Correction loss (°)	0.64 ± 2.2	0.68 ± 8.2	0.60 (U = 400)
CA (°)			
Pre-operation	22.9 ± 12.3	26.8 ± 12.8	0.26 (U = 359)
Post-operation	16.5 ± 8.2	19.8 ± 10.0	0.25 (U = 357)
At final follow-up	22.1 ± 11.2	23.3 ± 12.6	0.81 (U = 418)
Correction loss (°)	5.6 ± 4.8	3.5 ± 4.8	0.023* (U = 285)
Without a surgical-level fracture	5.0 ± 4.6	1.9 ± 3.1	0.0012* (U = 165)
AVBH (%)			
Pre-operation	58.0 ± 24.9	55.1 ± 31.4	0.38 (U = 376)
Post-operation	77.1 ± 21.8	83.6 ± 22.5	0.31 (U = 367)
At final follow-up	73.4 ± 20.5	82.6 ± 21.6	0.16 (U = 341)
Correction loss (%)	3.7 ± 8.1	1.1 ± 14.3	0.28 (U = 363)
PS loosening, n (%)	11 (39%)	5 (16%)	0.046*^,#^ (chi-square = 4.0, df = 1)
PS backout, n (%)	6 (21%)	0 (0%)	0.0084*^,##^
Adjacent vertebral fracture, n (%)	4 (14%)	6 (19%)	0.73^##^
Re-operation, n (%)	3 (11%)	1 (3%)	0.34^##^

Mechanical study

**Figure 4:**
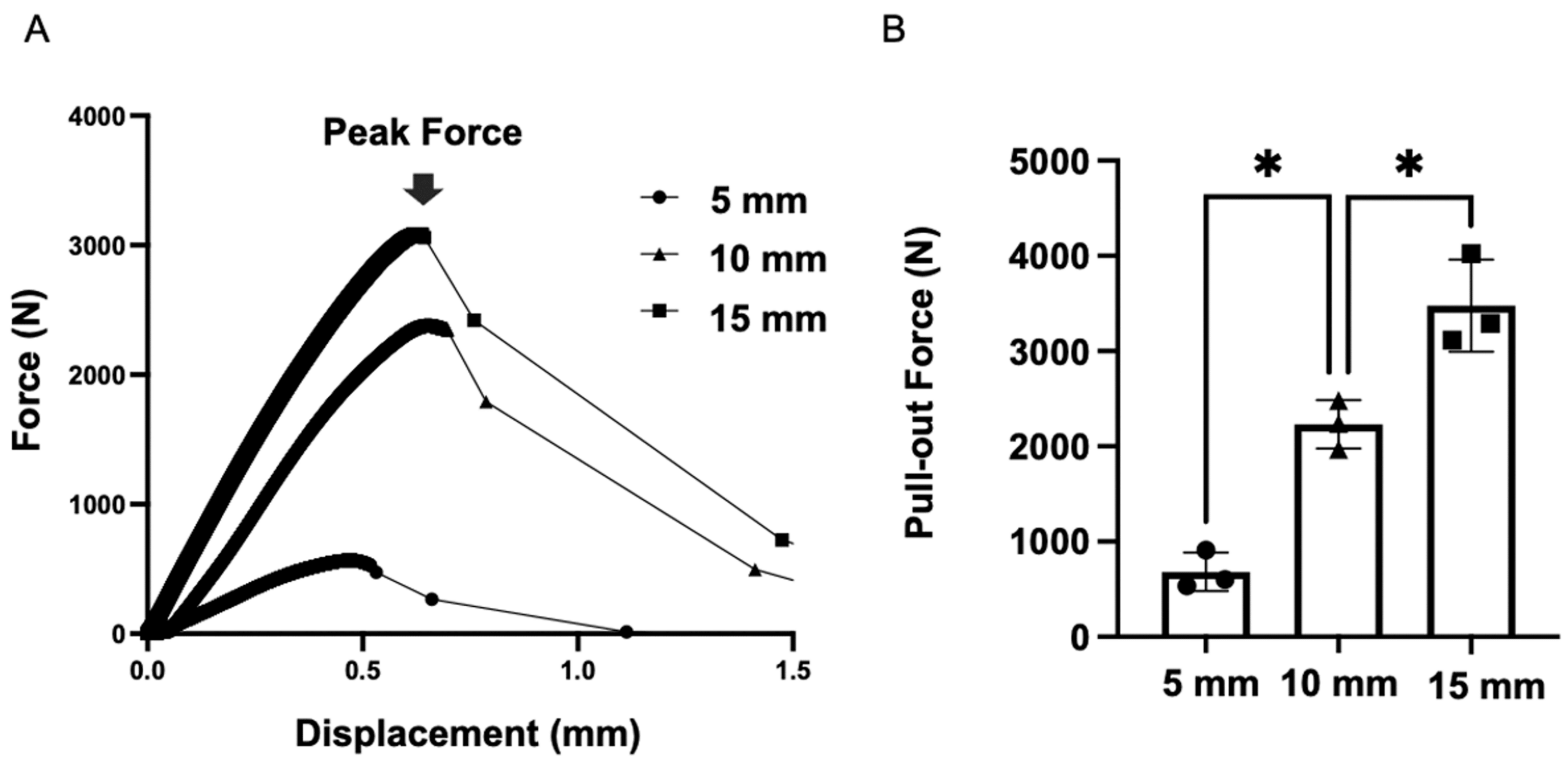
Axial pullout test (A) Force-displacement curve: The peak pullout force was determined as the point where the pullout force began to decrease on the curve. (B) Pullout force: The average pullout forces were as follows: 683 ± 164 N for a depth of 5 mm, 2231 ± 208 N for 10 mm, and 3477 ± 393 N for 15 mm. The pullout force increased significantly with the insertion depth into the cement (P = 0.003 and P = 0.009, respectively). Significance (*) was defined as P < 0.05.

The mean maximum pullout strengths for each insertion depth were as follows: 5 mm, 683 ± 164 N; 10 mm, 2231 ± 208 N; and 15 mm, 3477 ± 393 N. The pullout strength increased significantly in a stepwise manner with greater insertion depth (P = 0.003 and 0.009) (Figure 4). 

**Figure 5:**
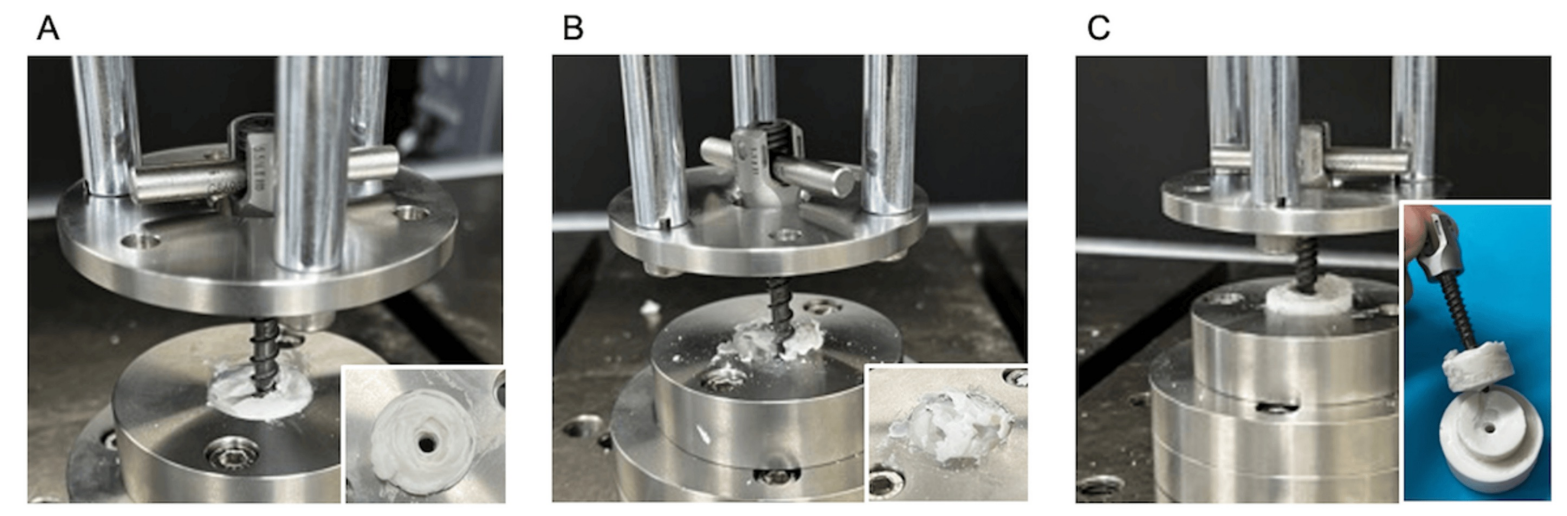
Point of failure For screws inserted to a depth of 5 mm, failure occurred at the screw-cement interface without cement breakage. In contrast, for screws inserted to depths of 10 and 15 mm, cement breakage occurred before screw pullout: (A) 5, (B) 10, and (C) 15 mm.

At 5-mm insertion, failure occurred at the cement-screw interface. However, at 10-mm insertion, failure occurred within the cement before screw-cement interface failure in two out of three cases, and at 15-mm insertion, cement failure occurred first in all three cases (Figure 5). Furthermore, all screws inserted to a depth of 15 mm could be removed using a screwdriver after testing.

## Discussion

This study evaluated the novel CCS technique, along with its clinical outcomes and mechanical strength. The key features of CCS include its ability to achieve high pullout strength by anchoring firmly within the cement while connecting the anterior and posterior elements. In addition, using tulip-type PS also enables linkage with adjacent PS to function as an IMS.

Regarding the pullout strength, the measured values were based on screw insertion depths, ranging from 683 to 3477 N. These strengths are comparable to or higher than those reported in the literature for PS inserted into osteoporotic human vertebrae (144-828 N) [14-19]. Although this study evaluated the pullout strength at the cement-screw interface, excluding contributions from cortical and cancellous bones, even a 5-mm insertion (approximately 1.5 threads) achieved an average strength of 683 N, which is comparable to that of PS in osteoporotic vertebrae. Sarzier et al. reported increased pullout strength with cement-augmented PS (576-1363 N) by injecting cement into screw holes in cadaver vertebrae compared with standard PS (289-765 N) [18]. Similarly, Chao et al. demonstrated that cementing into pilot holes (811 ± 274 N) was more effective than fenestrated screws (579 ± 249 N), emphasizing that the engagement of screw threads with cement enhances strength [14]. Our study achieved even higher pullout strengths than these cadaver studies. This difference may be attributed to the fact that our measurements targeted the cement-screw interface, whereas cadaver studies primarily reflect the cement-bone interface. In cadaver studies, the screw and cement were extracted as a single unit, indicating that the measured strength reflects the bond between the cement and bone. These findings indicate that in osteoporotic vertebrae, even minimal screw insertion of 5 mm into the cement can achieve higher pullout strength than at the cement-bone interface.

Clinically, combining BKP with two-level posterior fixation using CCS as IMS significantly reduced postoperative correction loss within the fixation range. Moreover, loosening and pullout of adjacent-level PS were effectively suppressed. Since Dick et al. introduced IMS in 1994, various biomechanical analyses have demonstrated their ability to enhance construct stiffness, reduce the anterior load on the fractured vertebra, and decrease stress on PS inserted into adjacent vertebrae [20-22]. A 2020 systematic review, which compared two-level posterior fixation with one above and one below (four-screw construct) versus IMS-assisted fixation (six-screw construct), demonstrated that it significantly reduced postoperative correction loss and implant failure, although IMS increased the operative time [23]. The reported correction loss of the CA in six-screw constructs ranged from 0° to 4° [24], which is comparable with the mean correction loss (3.5°) observed in this study using CCS. Furthermore, the previous studies included patients aged 35.3-46.4 years, whereas this study targeted an older population, with an average age of 77.4 years, highlighting the potential efficacy of CCS in improving fixation stability even in older patients.

Regarding BKP and IMS, a comparison between two-level posterior fixation with BKP and without BKP but with IMS revealed that the IMS group had significantly prevented kyphotic deformity [25]. Additionally, an FEM analysis of osteoporotic vertebrae comparing four different constructs, four- and six-screw constructs, with or without cement vertebroplasty, demonstrated that the combination of cement vertebroplasty and IMS in a six-screw construct provided the most robust construct and reduced stress on adjacent PS [26]. Furthermore, in a study involving patients with OVFs (average age of 58.2 years), the CA correction loss with a six-screw construct combined with vertebroplasty and IMS was 7.25° ± 4.85° [27]. Compared with the correction loss observed in this study (3.5° ± 4.8°) using CCS as an IMS for OVF treatment, the CCS group achieved significantly less correction loss (P = 0.0018). This superior outcome may be because of the longer screw insertion distance beyond the cement-reached area and the strong pullout strength caused by cement anchorage.

**Figure 6:**
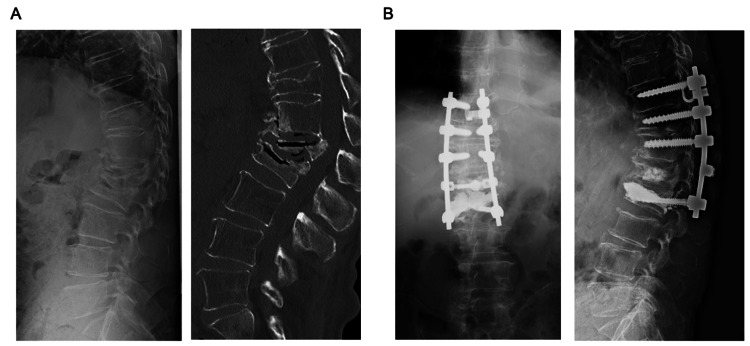
Future prospects: clinical case of a 73-year-old male Chief complaint: Inability to walk because of back and leg pain. (A) Preoperative imaging (X-ray and computed tomography): The L1 and L2 vertebral bodies exhibited pseudarthrosis with the presence of intravertebral vacuum phenomena. Bony fusion was observed in more than four vertebrae proximal to T12, confirming the findings of diffuse idiopathic skeletal hyperostosis. (B) Postoperative imaging (X-ray): Balloon kyphoplasty was performed at the L2 vertebral body, and cement-catching screws were inserted as a caudal anchor. No screw pullout was observed at six months postoperatively.

In this study, CCS were used primarily as IMS to connect the anterior and posterior elements and enhance the strength of short constructs. As a future prospect, we are considering the potential application of CCS in multiple fractures and adjacent vertebral fractures following fixation surgery by inserting CCS into the fractured vertebra and using them as fixation endpoints (Figure 6). This approach may allow the use of fractured vertebrae, traditionally unsuitable as anchors, as strong fixation anchors, potentially shortening the fixation range.

This study has several limitations. First, it was a retrospective study with a small sample size. Second, an ideal control group would include IMS with standard screws for a more direct comparison. Third, follow-up was limited to 6-12 months, and long-term outcomes remain unclear. Fourth, most patients were older, and detailed clinical assessments such as the visual analog scale and patient-reported outcome measures were challenging due to cognitive issues. Future studies are needed to overcome these limitations. Finally, CCS can only be placed on vertebrae with BKP, limiting its use to cases of compression fractures.

## Conclusions

We developed a novel screw insertion technique called CCS, which involves the insertion of screws after cement hardening during BKP to securely anchor into the cement. The biomechanical test demonstrated its strong pullout strength, and clinical outcomes were favorable. The use of CCS contributes to less invasive OVF treatment and helps reduce implant-related complications.
